# Rapid efficacy testing of cattle footbath disinfectants by flow cytometry: a laboratory-validated screening method complementary to EN 1656

**DOI:** 10.2478/jvetres-2026-0038

**Published:** 2026-07-18

**Authors:** Lucie Langová, Petr Kouřil, Vladimír Zmrhal, Lenka Hromádková, Miroslav Macháček, Libor Kalhotka, Zdeněk Havlíček

**Affiliations:** 1 Department of Animal Breeding, Animal Nutrition & Biochemistry, Department of Animal Protection and Welfare & Veterinary Public Health, University of Veterinary Sciences Brno, 61242 Brno, Czech Republic; 2 Department of Morphology, Physiology and Animal Genetics, Department of Food Technology, Department of Animal Breeding, Mendel University in Brno, 61300 Brno, Czech Republic; 3 Department of Food Technology, Department of Animal Breeding, Mendel University in Brno, 61300 Brno, Czech Republic; 4 Department of Animal Breeding, Mendel University in Brno, 61300 Brno, Czech Republic; 5 Unit of Biological Methods, Institute for State Control of Veterinary Biologicals and Medicines, 62100 Brno, Czech Republic; 6 Department of Animal Protection and Welfare & Veterinary Public Health, University of Veterinary Sciences Brno, 61242 Brno, Czech Republic

**Keywords:** bovine, fluorescent staining, hoof disinfection, membrane integrity, prophylaxis

## Abstract

**Introduction:**

Disinfectants are essential for preventing infectious diseases in livestock, particularly in cattle footbath applications. Conventional culture-based methods for assessing bactericidal efficacy are reliable but time-consuming, leaving the need unmet for rapid laboratory-based screening approaches.

**Material and Methods:**

Thirteen commercially available cattle footbath disinfectants were evaluated using the EN 1656 dilution–neutralisation method and flow cytometry (FCM) with propidium iodide (PI) staining. A binary logistic regression model was developed to relate the proportion of PI^+^ cells to bactericidal efficacy.

**Results:**

Disinfectants containing iodine or iodide, organic acids, hydrogen peroxide, quaternary ammonium compounds, glutaraldehyde or alcohol showed high bactericidal efficacy, whereas those with zinc sulphate were ineffective. The predictive model demonstrated good performance (AUC = 0.787) and identified an optimal threshold of approximately 80% PI^+^ cells, yielding 85.47% sensitivity and 76.92% specificity. Agreement between FCM and the EN 1656 method was high, with 7.69% inconsistent classifications.

**Conclusion:**

Flow cytometry represents a rapid and efficient laboratory-based screening method with the potential to complement culture-based approaches for evaluating bovine footbath disinfectant efficacy.

## Introduction

Poor hygiene and persistently moist conditions on livestock farms are major drivers of infectious hoof diseases, which substantially compromise dairy cattle welfare and productivity (1, 26). Disinfectant footbaths are a widely applied and effective strategy for reducing the prevalence and transmission of infectious hoof diseases, although they do not affect non-infectious claw lesions associated with claw horn disruption.

A wide range of disinfectants is currently used in cattle footbaths, differing in their active ingredients, which include organic acids, quaternary ammonium compounds, iodine, formaldehyde and metal salts. Among these, formaldehyde and copper sulphate remain the most commonly applied disinfectants in practice (6). European legislation requires that veterinary biocides demonstrate proven bactericidal efficacy under the conditions recommended by the manufacturer, and the demonstration is most commonly through standardised culture-based methods such as the European standard EN 1656 (8).

While culture-based methods provide a robust reference for disinfectant efficacy testing, they are inherently time-consuming and limited to bacteria that can be cultivated under laboratory conditions. Enumeration typically requires at least 24 h of incubation and this may even exceed 48 h for anaerobic organisms (3). As noted by McFeters (17), such methods are not applicable to non-cultivable bacteria, including *Treponema* spp., which are strongly associated with digital dermatitis in cattle. Consequently, culture-based approaches may underestimate the actual number of viable bacterial cells present in disinfected samples (21).

Flow cytometry (FCM) is a rapid and sensitive technique that enables the differentiation of bacterial physiological states based on membrane integrity and metabolic activity (21, 27). By using fluorescent dyes such as propidium iodide (PI), flow cytometry facilitates the assessment of bactericidal and bacteriostatic effects and the detection of viable but non-culturable (VBNC) cells (2). Compared to traditional colony counting, this approach offers a substantial reduction in analysis time while providing quantitative insight into bacterial viability (2, 10).

The objective of this study was to evaluate the bactericidal efficacy of commercially available cattle footbath disinfectants under laboratory conditions by comparing FCM with the standardised EN 1656 dilution–neutralisation method, and to assess whether flow cytometry can serve as a rapid laboratory-based screening approach for disinfectant efficacy in veterinary practice.

## Material and Methods

### Tested bacteria

The bacteria used for disinfection testing were as follows:

 i. Gram-positive *Staphylococcus aureus* (CCM 2022)

 ii. Gram-positive *Enterococcus hirae* (CCM 4533)

 iii. Gram-negative *Pseudomonas aeruginosa* (CCM 7930)

 iv. Gram-negative *Proteus hauseri* (CCM 7011), formerly *Proteus vulgaris* genomospecies 3 (18)

These microorganisms were from the Czech Collection of Microorganisms (CCM) at Masaryk University (Brno, Czech Republic.

### Disinfectants

Thirteen commercially available disinfectants intended for use in cattle footbaths were included in the study (Table 1). All experiments were performed using the manufacturer’s recommended working concentration (MWC). To further characterise efficacy profiles, the dilution–neutralisation method was additionally performed at reduced concentrations (typically 50% and 25% of the MWC) to simulate potential dilution effects under farm conditions. The lowest concentration achieving bactericidal efficacy in the dilution-neutralisation test was used for FCM. Unless stated otherwise, results refer to the MWC to allow direct comparison between methods.

**Table 1.** Cattle footbath disinfectants evaluated by flow cytometry as a complementary method to European standard EN 1656 dilution–neutralisation

Disinfectant	Main antimicrobial agent	Characteristics and spectrum of activity	Composition	Concentration (%)
A	Oxidising compounds	crystalline, blue, odourless, pH 3.5–4.5 (at 20°C); unspecified spectrum of activity	CuSO_4_(pentahydrate)	5
B	Oxidising compounds	crystalline, white, odourless, pH 4–6 (at 20°C), unspecified spectrum of activity	ZnSO_4_(heptahydrate)	10
C (no longer available)	Oxidising compounds	crystalline, green, unspecified spectrum of activity	a mixture of organic acids, copper and zinc salts	1
D	Oxidising compounds	liquid, brown, pH 2 (at 20°C), bactericidal, fungicidal, sporicidal and virucidal	sodium iodide ≤7%, phosphoric acid ≤5%, sulfuric acid ≤5%, aqueous solution of sodium salt of fatty alcohol ethoxy sulphate ≤5%, iodine ≤3%	1
E (no longer available in the original composition)	Chelating complexes	No longer available in the original composition	copper-chelating complex 55%, zinc-chelating complex 55%, N-(3-aminopropyl)-N-dodecyl propane-1,3-diamine	1
F	Oxidising compounds	liquid, dark green, solvent odour, pH 2.6 (at 20°C), unspecified spectrum of activity	L – lactic acid (2-hydroxy propionic acid) 15–30%, citric acid 1–5%, octanoic acid 1–5%, 2(2-butoxy ethoxy) ethanol 1–5%, anionic surfactants 1–5%	3
G	Oxidising compounds	liquid, green, peroxide odour, pH 4.8 (at 20°C), bactericidal	hydrogen peroxide 11%, alkyl (C12–16) dimethyl benzyl ammonium chloride 2.8%, didecyldimethylammonium chloride 2%, guanidine, N, N'''-1,3-propanediylbis-, N-coco alkyl derivatives, diacetates <5%, ethoxylated (EO 8) C10 alcohol <5%, propane-2ol 1.5%	1
H	Aldehydes	liquid, colourless, pungent odour, pH 2.8–4.0 (at 20°C), unspecified spectrum of activity	formaldehyde 36–38%	2
I	Aldehydes, alcohols	liquid, clear orange/red, pungent glutaraldehyde odour, pH 1.7, bactericidal, fungicidal and virucidal	glutaraldehyde 10–15%, ethoxylated alcohol C12–C15 5–10%, dodecyl-(dimethyl)ammonium chloride 3–5%, isopropanol 1–3%, methanol 0.1–1%, phosphoric acid 0.1–1%	5
J	Aldehydes	liquid, green, characteristic odour, pH 1.1–1.7, unspecified spectrum of activity	glutaraldehyde <10%, alkyldimethyl-benzylammonium chloride <5%, copper sulfate pentahydrate <5%, zinc sulphate <5%, phosphoric acid <3%	1
K	Aldehydes	liquid, colour, aldehyde odour, pH 2.5 (at 20°C), biocidal	glutaraldehyde 10–25%, alkyldimethyl-benzylammonium chloride 1–5%, copper sulphate 1–5%, aluminium sulphate 1–5%	2
L	Anionic detergent (foaming agent), Alcohols	liquid, blue-green, indistinct odour, pH 3.2–3.6 (at 20°C), broad-spectrum bactericidal, fungicidal	sodium laureth sulphate <8.0%, glyoxal ≤8.0%, ethane-1,2diol 0.5%, glutaral (1,5-pentandial) ≤2.5%, oxirane, 2-methyl-, polymer with oxirane, mono (2-propyheptyl) ether ≤2.8%, copper sulphate <1.5%	5
M	Oxidising compounds	liquid, clear dark brown, weak iodine odour, surface active, pH 0, bactericidal, fungicidal, virucidal and sporicidal	alcohol (C9–11) ethoxylate (8EO) 20–25%, sulfuric acid 5–10%, orthophosphoric acid 5–10%, iodine 1–3%	0.5

1 The concentration column indicates the manufacturer’s recommended working concentration for practical on-farm use. It was used for all flow cytometry and dilution–neutralisation experiments. Each disinfectant was additionally tested in dilution–neutralisation at two lower concentrations (typically 50% and 25% of the listed value) to assess a broader efficacy range

### Dilution–neutralisation method

Bactericidal efficacy was assessed using a quantitative suspension test according to EN 1656. Briefly, bacterial suspensions were exposed to disinfectants for 5 min at 21°C, after which validated neutralising agents were added which were selected according to disinfectant composition (Table 2). Surviving bacteria were enumerated by plating on tryptone soy agar and incubating for 24 h at 37°C. All experiments were performed in triplicate. Bactericidal activity was expressed as logarithmic reduction (LogR), this being calculated as the difference between the base-10 logarithm of the initial bacterial count (N_0_) and that after exposure (N_a_) (LogR = log_10_(N_0_) – log_10_(N_a_). The variable N_0_ is the number of viable bacteria (in colony-forming units (CFU)/mL) in the initial control suspension, and N_a_ is the number of viable bacteria (CFU/mL) after 5 min of exposure to the disinfectant). Values of LogR ≥ 5 are considered bactericidal according to EN 1656.

**Table 2.** Suitable neutralisers for cattle footbath disinfectants according to their composition

Antimicrobial ingredient	Neutraliser
Quaternary ammonium compounds and fatty amines; amphoteric compounds; biguanides and similar compounds; alcohols; phenolic and related compounds: orthophenylphenol, phenoxyethanol, triclosan, phenyl ethanol, etc.; and anilides	Polysorbate 80, 30g/L+ saponin 30 g/L + lecithin 3 g/L
Oxidising compounds (chlorine, iodine, hydrogen peroxide, peracetic acid, hypochlorites, etc.)	Sodium thiosulphate 3 g/L+ polysorbate 80, 30 g/L + lecithin 3 g/L
Aldehydes	Polysorbate 80, 30 g/L+ lecithin 3 g/L + L-histidine 1 g/l or glycine 1 g/L

### Flow cytometry methodology

Bacterial strains were cultured in TSB for 24 h. Aliquots of bacterial suspension (20 mL; 1.5 × 10^8^ – 5 × 10^8^ CFU/mL, approximately 0.5 McFarland) were transferred to sterile 50-mL centrifuge tubes and centrifuged at 3,000 rpm for 5 min. The resulting cell pellets were resuspended in 20 mL of sterile saline solution. An equal volume (20 mL) of disinfectant was added, and samples were incubated for 5 min at room temperature. The reaction was stopped by centrifugation at 3,000 rpm for 5 min, and the supernatant was discarded. To remove residual disinfectant and prevent carry-over effects, cell pellets were washed at least twice with 20 mL of Ca^2+^-free and Mg^2+^-free PBS (Biosera, Cholet, France), with the number of washing steps adjusted according to disinfectant type. Disinfectant concentrations used for FCM analysis corresponded to the lowest effective concentrations determined by the dilution–neutralisation method. For viability assessment, 5 μL of PI (Invitrogen/Thermo Fisher Scientific, Eugene, OR, USA) was added to 1 mL of bacterial suspension. Untreated samples served as viability controls. All samples were prepared and analysed in triplicate using a BriCyte E6 flow cytometer (Mindray, Shenzhen, China) and processed within 30 min of disinfectant exposure to minimise post-treatment physiological changes.

Bacterial populations were identified by forward and side-scatter signals, and membrane-compromised (non-viable) cells were defined as PI^+^ based on fluorescence intensity in the phycoerythin channel. Gating strategies distinguishing viable (PI^-^) and non-viable (PI^+^) cells were established using untreated controls. Representative flow cytometric profiles for *Staphylococcus aureus* CCM 2022 before and after disinfectant exposure are shown in Fig. 1

**Fig. 1.**
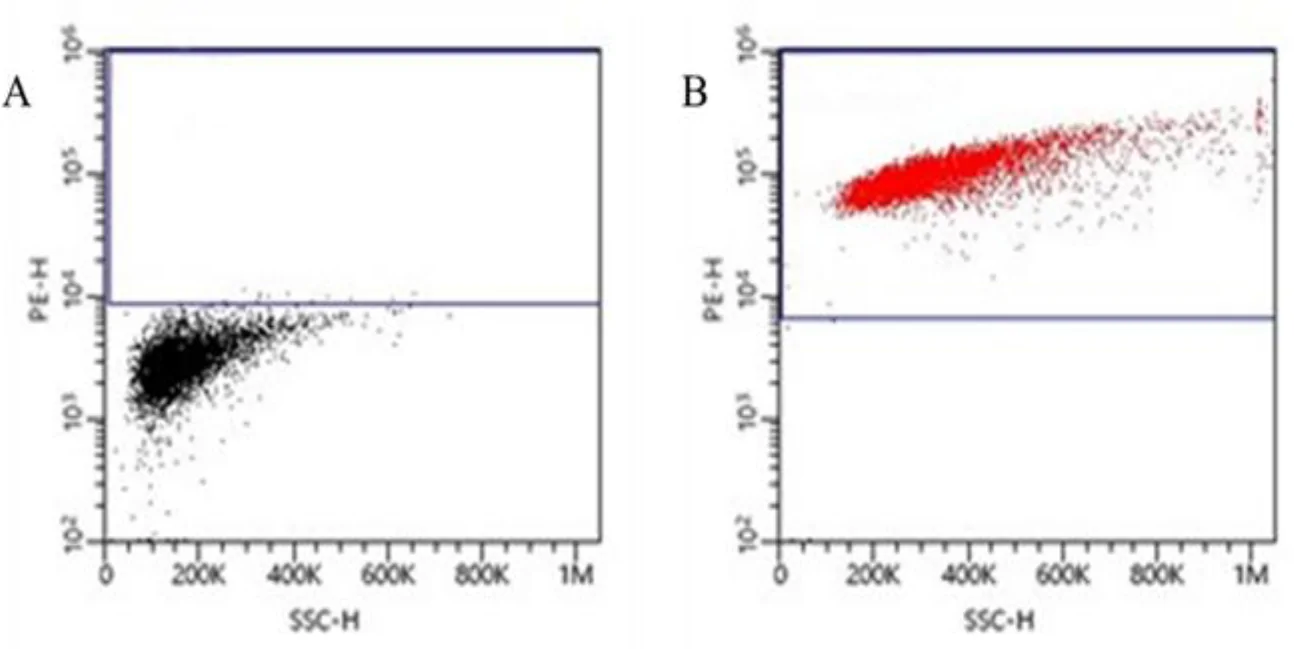
Flow cytometry analysis of *Staphylococcus aureus* viability before and after disinfectant treatment. (A) Dot plot of an untreated control sample showing a predominantly viable propidium-iodide (PI)^–^ population characterised by low fluorescence in the phycoerythin (PE) channel. (B) Dot plot of a sample treated with an effective disinfectant, demonstrating a shift to a non-viable PI^+^ population with high PE fluorescence. The horizontal line in both plots represents the threshold used to distinguish between PI^-^ and PI^+^ cells. PE-H – PE pulse height; SSC-H – side-scatter pulse height

### Statistical evaluation

Statistical analyses were performed using GraphPad Prism v. 8.0.1 (GraphPad Software, Boston, MA, USA). Data normality was assessed using the Shapiro–Wilk test. The assumption of homogeneity of variances was formally tested using Levene’s test. In cases where this assumption was violated, data transformation or Welch’s ANOVA was applied to ensure the robustness of the results. Differences in disinfectant efficacy were evaluated by two-way ANOVA with disinfectant and bacterial species as factors, followed by Tukey’s multiple comparison test. Statistical significance was set at P-value < 0.05.

To establish a quantitative efficacy threshold for flow cytometry, a univariate binary logistic regression model was constructed using SAS software (SAS Institute, Cary, NC, USA), with bactericidal classification as the outcome variable and the percentage of PI^+^ cells as the predictor. Model performance was evaluated using ROC curve analysis, and the optimal cutoff value was determined by maximising the Youden index.

## Results

### Efficacy assessment by dilution–neutralisation method

Bactericidal efficacy was assessed using such a method in accordance with EN 1656, where a bactericidal effect is defined as a LogR ≥ 5. At the MWCs, the majority of tested disinfectants demonstrated pronounced bactericidal activity against the selected bacterial strains (Table 3).

**Table 3.** Comparison of bactericidal efficacy of the tested cattle footbath disinfectants assessed by the dilution–neutralisation method and flow cytometry for individual disinfectant–bacterium combinations

Disinfectant	*Staphylococcus aureus* PI^+^ (%)	*Staphylococcus aureus* LogR	*Enterococcus hirae* PI^+^ (%)	*Enterococcus hirae* LogR	*Pseudomonas aeruginosa* PI^+^ (%)	*Pseudomonas aeruginosa* LogR	*Proteus hauseri* PI^+^ (%)	*Proteus hauseri* LogR
A	79.36	3.87	13.02	3.73	93.03	≥ 5.19	27.18	≥ 5.27
B	8.62	4.12	8.39	4.35	24.58	4.32	27.17	3.82
C	99.30	3.87	70.18	3.73	90.98	≥ 5.19	95.02	≥ 5.19
D	99.80	≥ 5.45	99.19	≥ 5.48	98.98	≥ 5.12	98.43	≥ 5.20
E	9.06	3.03	11.6	4.01	35.04	≥ 5.26	43.55	3.78
F	98.42	≥ 5.15	99.83	≥ 5.32	98.50	≥ 5.18	99.35	≥ 5.59
G	99.68	≥ 5.43	99.48	≥ 5.65	97.24	≥ 5.62	99.22	≥ 5.12
H	35.45	4.58	5.25	3.73	85.68	≥ 5.19	69.34	≥ 5.27
I	99.90	≥ 5.40	99.52	≥ 5.75	99.54	≥ 6.18	98.20	≥ 5.87
J	99.71	≥ 5.12	95.90	≥ 5.15	98.74	≥ 5.05	99.08	≥ 5.60
K	98.90	≥ 5.40	99.76	≥ 5.75	87.81	≥ 6.18	80.99	≥ 5.87
L	99.72	≥ 5.40	99.53	≥ 5.75	98.62	≥ 6.18	98.92	≥ 5.87
M	99.71	≥ 5.57	99.57	≥ 5.73	99.89	≥ 5.18	99.77	≥ 5.22

1 A – CuSO_4_4 (pentahydrate) at 5%; B – ZnSO_4_ (heptahydrate) at 10%; C – mixture of organic acids, copper and zinc salts at 1%; D – sodium iodide ≤7%, phosphoric acid ≤5%, sulfuric acid ≤5%, aqueous solution of sodium salt of fatty alcohol ethoxy sulphate ≤5%, iodine ≤3% at 1%; E – copper-chelating complex 55%, zinc-chelating complex 55%, N-(3-aminopropyl)-N-dodecyl propane-1,3-diamine at 1%; F – lactic acid (2-hydroxy propionic acid) 15–30%, citric acid 1–5%, octanoic acid 1–5%, 2(2-butoxy ethoxy) ethanol 1–5%, anionic surfactants 1–5% at 3%; G – hydrogen peroxide 11%, alkyl (C12-–16) dimethyl benzyl ammonium chloride 2.8%, didecyldimethylammonium chloride 2%, guanidine, N, N'''-1,3-propanediylbis-, N-coco alkyl derivatives, diacetates <5%, ethoxylated (EO 8) C10 alcohol <5%, propane-2ol 1.5% at 1%; H – formaldehyde 36–38% at 2%; I – glutaraldehyde 10–15%, ethoxylated alcohol C12–C15 5–10%, didecyldodecyl-(dimethyl)ammonium chloride 3–5%, isopropanol 1–3%, methanol 0.1–1%, phosphoric acid 0.1–1% at 5%; J – glutaraldehyde <10%, alkyldimethyl-benzylammonium chloride <5%, copper sulfate pentahydrate <5%, zinc sulphate <5%, phosphoric acid <3% at 1%; K – glutaraldehyde 10–25%, alkyldimethyl-benzylammonium chloride 1–5%, copper sulphate 1–5%, aluminium sulphate 1–5% at 2%; L – sodium laureth sulphate <8.0%, glyoxal ≤8.0%, ethane-1,2diol 0.5%, glutaral (1,5-pentandial) ≤2.5%, oxirane, 2-methyl-, polymer with oxirane, mono (2-propyl heptyl) ether ≤2.8%, copper sulphate <1.5% at 5%; M – alcohol (C9–11) ethoxylate (8EO) 20–25%, sulfuric acid 5–10%, orthophosphoric acid 5–10%, iodine 1–3% at 0.5%. Bactericidal activity was defined as a logarithmic reduction (LogR) ≥ 5 according to EN 1656 for the culture-based method and as ≥80% propidium iodide-positive (PI^+^) cells for flow cytometry

Disinfectants D, F, G and I–M consistently achieved LogR values ≥ 5 against all four tested microorganisms, indicating broad-spectrum bactericidal efficacy. In contrast, zinc sulphate (B) failed to reach the bactericidal threshold for any of the tested bacteria and was therefore classified as non-bactericidal across all strains. Several disinfectants exhibited microorganism-dependent efficacy. Copper sulphate (A); an organic acid, copper and zinc salt mixture (C); and formaldehyde (H) achieved bactericidal activity against *Pseudomonas aeruginosa* and *Proteus hauseri* but did not reach LogR ≥ 5 against *Staphylococcus aureus* or *Enterococcus hirae*. Copper-chelating and zinc-chelating complexes (E) demonstrated bactericidal activity exclusively against *P. aeruginosa* while remaining ineffective against the other tested strains. Multiple disinfectants failed to meet the EN 1656 bactericidal criterion for bactericidal effectiveness against *E. hirae*, which appeared to be the most resistant species in the culture-based assay.

### Efficacy assessment by flow cytometry

Flow cytometry analysis revealed statistically significant differences in bactericidal efficacy among the tested disinfectants for each bacterial strain (P-value < 0.05) (Fig. 2). Overall, disinfectants D, F, G and I–M induced extensive membrane damage, as indicated by high proportions of PI^+^ cells typically exceeding 95%. Disinfectants B and E exhibited significantly lower bactericidal efficacy against *Staphylococcus aureus* compared to the remaining preparations, with reduced proportions of PI^+^ cells. A moderate reduction in efficacy was also observed for disinfectants A and H. Against *Pseudomonas aeruginosa*, disinfectants B and E again showed insufficient bactericidal effects, whereas most other preparations’ use resulted in high PI^+^ cell proportions.

**Fig. 2.**
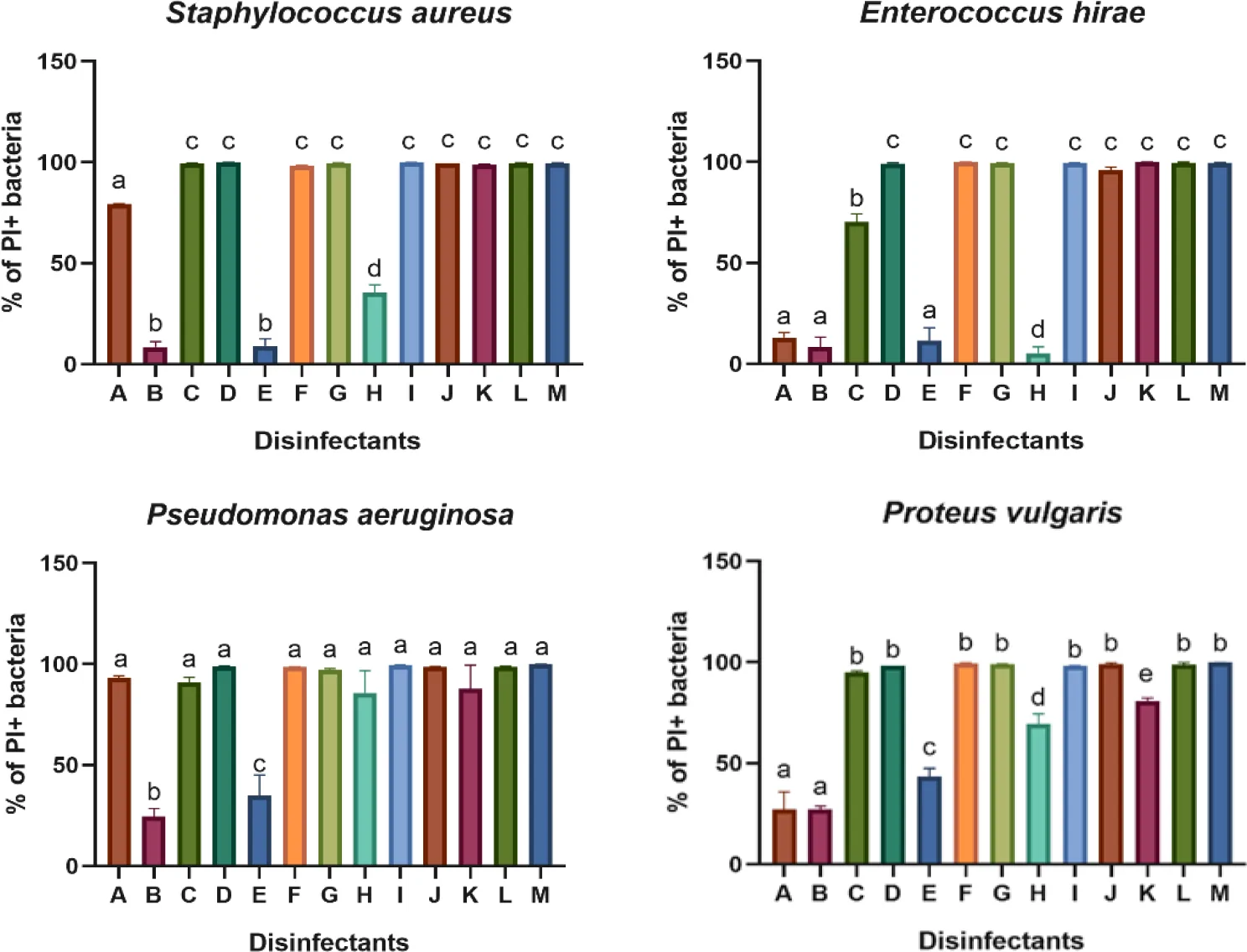
Comparison of the effectiveness of cattle footbath disinfectants against individual bacteria as indicated by propidine-iodide (PI)-positive cell proportions. A – CuSO_4_4 (pentahydrate) at 5%; B – ZnSO_4_ (heptahydrate) at 10%; C – mixture of organic acids, copper and zinc salts at 1%; D – sodium iodide ≤7%, phosphoric acid ≤5%, sulfuric acid ≤5%, aqueous solution of sodium salt of fatty alcohol ethoxy sulphate ≤5%, iodine ≤3% at 1%; E – copper-chelating complex 55%, zinc-chelating complex 55%, N-(3-aminopropyl)-N-dodecyl propane-1,3-diamine at 1%; F – lactic acid (2-hydroxy propionic acid) 15–30%, citric acid 1–5%, octanoic acid 1–5%, 2(2-butoxy ethoxy) ethanol 1–5%, anionic surfactants 1–5% at 3%; G – hydrogen peroxide 11%, alkyl (C12-–16) dimethyl benzyl ammonium chloride 2.8%, didecyldimethylammonium chloride 2%, guanidine, N, N'''-1,3-propanediylbis-, N-coco alkyl derivatives, diacetates <5%, ethoxylated (EO 8) C10 alcohol <5%, propane-2ol 1.5% at 1%; H – formaldehyde 36–38% at 2%; I – glutaraldehyde 10–15%, ethoxylated alcohol C12–C15 5–10%, didecyldodecyl-(dimethyl)ammonium chloride 3–5%, isopropanol 1–3%, methanol 0.1–1%, phosphoric acid 0.1–1% at 5%; J – glutaraldehyde <10%, alkyldimethyl-benzylammonium chloride <5%, copper sulfate pentahydrate <5%, zinc sulphate <5%, phosphoric acid <3% at 1%; K – glutaraldehyde 10–25%, alkyldimethyl-benzylammonium chloride 1–5%, copper sulphate 1–5%, aluminium sulphate 1–5% at 2%; L – sodium laureth sulphate <8.0%, glyoxal ≤8.0%, ethane-1,2diol 0.5%, glutaral (1,5-pentandial) ≤2.5%, oxirane, 2-methyl-, polymer with oxirane, mono (2-propyl heptyl) ether ≤2.8%, copper sulphate <1.5% at 5%; M – alcohol (C9–11) ethoxylate (8EO) 20–25%, sulfuric acid 5–10%, orthophosphoric acid 5–10%, iodine 1–3% at 0.5%. Columns with different letters present significantly different values P-value < 0.05)

Disinfectant action was generally weakest against the Gram-positive bacterium *Enterococcus hirae* in the FCM assay. Disinfectants A, B and E produced significantly lower PI^+^ proportions compared to other disinfectants, and H exhibited the weakest bactericidal effect against this strain, differing significantly from disinfectant C. The greatest variability in bactericidal efficacy was observed against *Proteus hauseri*, with significant differences detected among disinfectants A, B, E, H and K. Across all bacterial strains, disinfectant M showed the highest overall efficacy, with a mean of 99.73% PI^+^ cells, followed by disinfectants I (99.29%) and F (99.03%).

### Comparative analysis and development of the predictive model

A direct comparison of bactericidal classification obtained by the dilution–neutralisation method and FCM is made in Table 4. Bactericidal activity was defined as LogR ≥ 5 for the culture-based method and ≥ 80% PI^+^ cells for FCM.

**Table 4.** Analysis of outcomes of bactericidal effect testing of 13 cattle footbath disinfectants comparing dilution–neutralisation (first outcome shown) with flow cytometry (second outcome shown)

Disinfectant	*Staphylococcus aureus* CCM 2022	*Enterococcus* CCM 4533	*hirae Pseudomonas* CCM 7930	*aeruginosa Proteus* CCM 7011	*hauseri* Overall agreement
A	– / –	– / –	+ / +	+ / –	3/4 (75%)
B	– / –	– / –	– / –	– / –	4/4 (100%)
C	– / +	– / –	+ / +	+ / +	3/4 (75%)
D	+ / +	+ / +	+ / +	+ / +	4/4 (100%)
E	– / –	– / –	+ / –	– / –	3/4 (75%)
F	+ / +	+ / +	+ / +	+ / +	4/4 (100%)
G	+ / +	+ / +	+ / +	+ / +	4/4 (100%)
H	– / –	– / –	+ / +	**+ / –**	3/4 (75%)
I	+ / +	+ / +	+ / +	+ / +	4/4 (100%)
J	+ / +	+ / +	+ / +	+ / +	4/4 (100%)
K	+ / +	+ / +	+ / +	+ / +	4/4 (100%)
L	+ / +	+ / +	+ / +	+ / +	4/4 (100%)
M	+ / +	+ / +	+ / +	+ / +	4/4(100%)

1 + / + – bactericidal by both methods; − / − – non-bactericidal by both methods; + / − – bactericidal by dilution–neutralisation method only; − / + – bactericidal by flow cytometry only; CCM – Czech Collection of Microorganisms; A – CuSO_4_4 (pentahydrate) at 5%; B – ZnSO_4_ (heptahydrate) at 10%; C – mixture of organic acids, copper and zinc salts at 1%; D – sodium iodide ≤7%, phosphoric acid ≤5%, sulfuric acid ≤5%, aqueous solution of sodium salt of fatty alcohol ethoxy sulphate ≤5%, iodine ≤3% at 1%; E – copper-chelating complex 55%, zinc-chelating complex 55%, N-(3-aminopropyl)-N-dodecyl propane-1,3-diamine at 1%; F – lactic acid (2-hydroxy propionic acid) 15–30%, citric acid 1–5%, octanoic acid 1–5%, 2(2-butoxy ethoxy) ethanol 1–5%, anionic surfactants 1–5% at 3%; G – hydrogen peroxide 11%, alkyl (C12-–16) dimethyl benzyl ammonium chloride 2.8%, didecyldimethylammonium chloride 2%, guanidine, N, N'''-1,3-propanediylbis-, N-coco alkyl derivatives, diacetates <5%, ethoxylated (EO 8) C10 alcohol <5%, propane-2ol 1.5% at 1%; H – formaldehyde 36–38% at 2%; I – glutaraldehyde 10–15%, ethoxylated alcohol C12–C15 5–10%, didecyldodecyl-(dimethyl)ammonium chloride 3–5%, isopropanol 1–3%, methanol 0.1–1%, phosphoric acid 0.1–1% at 5%; J – glutaraldehyde <10%, alkyldimethyl-benzylammonium chloride <5%, copper sulfate pentahydrate <5%, zinc sulphate <5%, phosphoric acid <3% at 1%; K – glutaraldehyde 10–25%, alkyldimethyl-benzylammonium chloride 1–5%, copper sulphate 1–5%, aluminium sulphate 1–5% at 2%; L – sodium laureth sulphate <8.0%, glyoxal ≤8.0%, ethane-1,2diol 0.5%, glutaral (1,5-pentandial) ≤2.5%, oxirane, 2-methyl-, polymer with oxirane, mono (2-propyl heptyl) ether ≤2.8%, copper sulphate <1.5% at 5%; M – alcohol (C9–11) ethoxylate (8EO) 20–25%, sulfuric acid 5–10%, orthophosphoric acid 5–10%, iodine 1–3% at 0.5%

Overall, a high level of agreement between the two methods was observed. Out of 52 disinfect–antbacterium combinations, 48 (92.3%) yielded consistent classifications of bactericidal or non-bactericidal activity. Complete agreement across all tested bacterial strains was observed for disinfectants B, D, F, G and I–M. Nevertheless, several discordant results were identified. Disinfectant C was classified as non-bactericidal against *S. aureus* based on the culture-based assay (LogR = 3.87), while FCM indicated that it caused extensive membrane damage, with 99.30% PI^+^ cells. Conversely, disinfectants A and H were classified as bactericidal against *P. hauseri* according to LogR values, despite relatively low PI^+^ cell proportions detected by FCM. Similarly, disinfectant E achieved bactericidal activity against *P. aeruginosa* based on LogR values, but this was not supported by a correspondingly high proportion of PI^+^ cells.

The relationship between LogR values and PI^+^ cell proportions, including the identified discordant classifications, is visualised in Fig. 3. Most observations clustered within concordant bactericidal or non-bactericidal regions, while discordant results were limited to a small number of data points located near the applied classification thresholds.

**Fig. 3.**
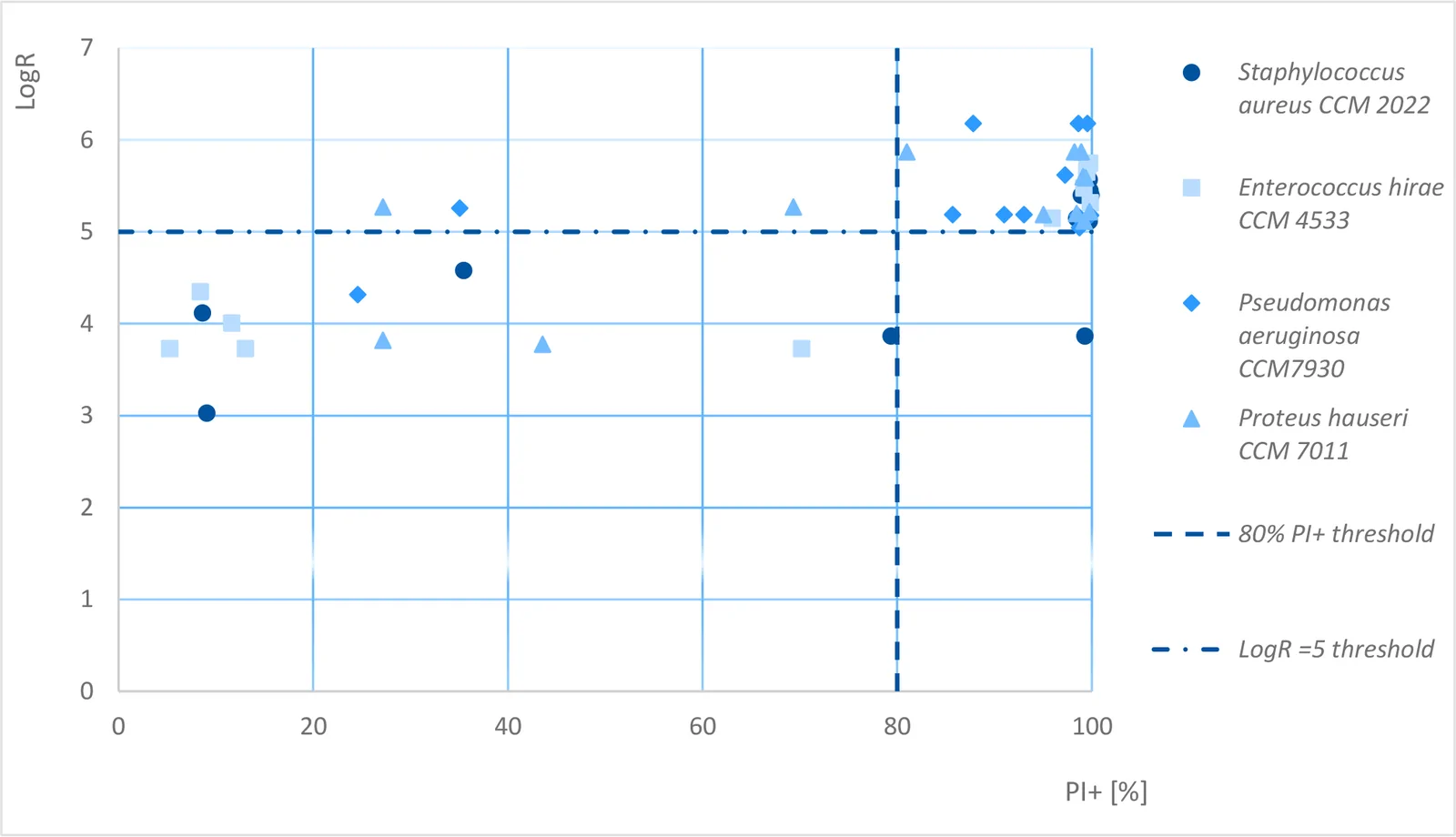
Scatter plot comparing flow cytometry (% PI^+^ cells) and culture-based (LogR) assessment of the bactericidal efficacy of 13 cattle footbath disinfectants. Dashed lines indicate classification thresholds for bactericidal activity (80% PI^+^ cells and LogR = 5). Each point represents one disinfectant–bacterium combination

### Development of the predictive model for flow cytometry-based classification

To establish a quantitative threshold for interpreting flow cytometry results, a binary logistic regression model was constructed using the percentage of cells as a predictor of bactericidal classification as determined by the dilution–neutralisation method. The model revealed a highly significant association between PI^+^ cell proportion and bactericidal classification (P-value < 0.0001), with an odds ratio of 1.039 (95% CI 1.027–1.052).

Analysis of the ROC curve demonstrated good discriminatory power, yielding an AUC of 0.787 (Fig. 4). Optimisation using the Youden index (0.62393) identified an optimal probability cutoff of 0.8091, corresponding to approximately 80.01% PI^+^ cells. At this threshold, the model achieved a sensitivity of 85.47% and a specificity of 76.92% for predicting bactericidal efficacy.

**Fig. 4.**
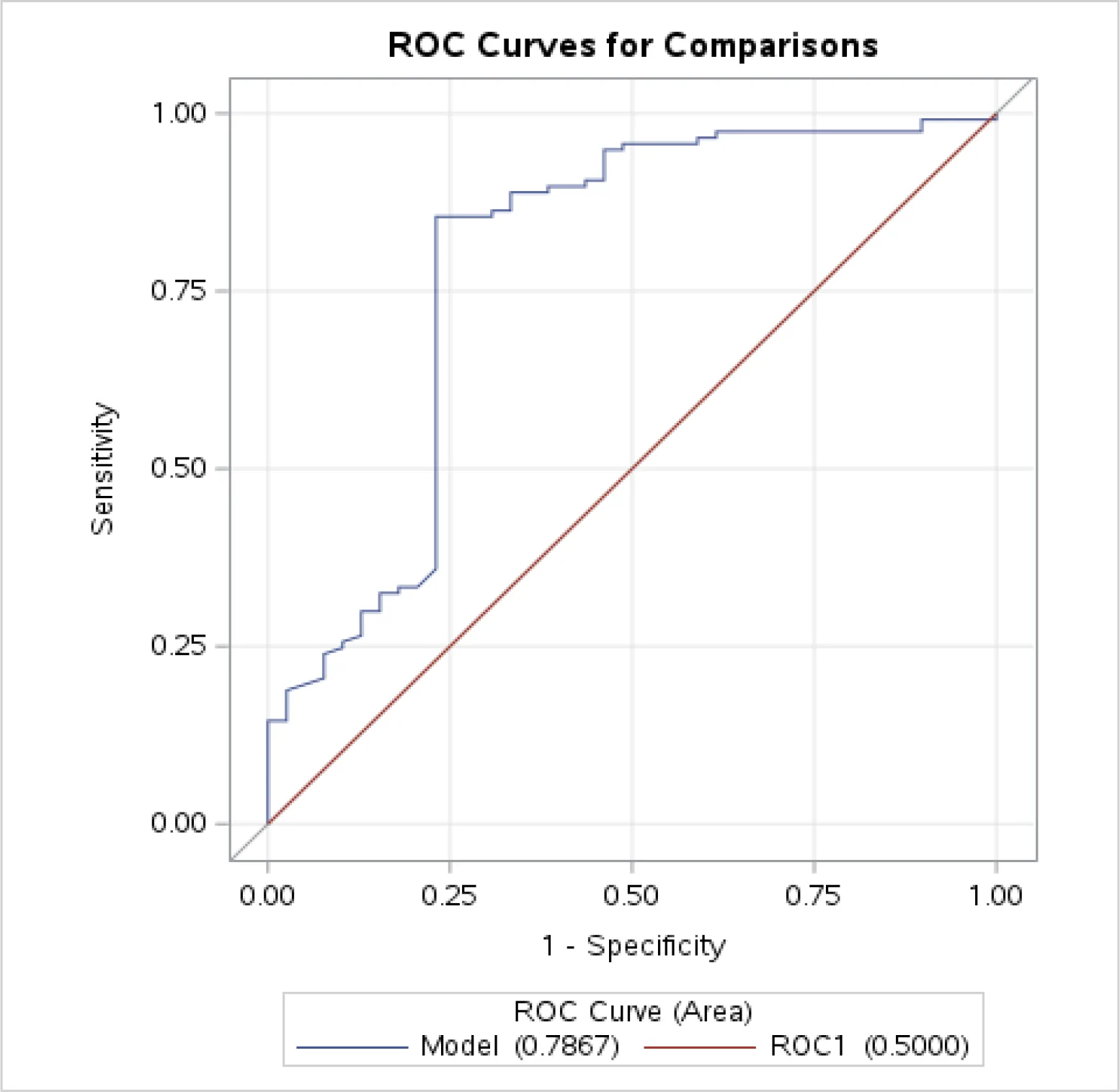
ROC curve for predicting the bactericidal efficacy of a disinfectant

## Discussion

The rigorous, evidence-based evaluation of chemical disinfectants is essential given the substantial variability in efficacy observed among the 13 commercial footbath products tested in this study. Direct comparison of a rapid FCM assay with the standardised culture-based EN 1656 method demonstrated a high level of agreement between the two approaches, indicating that FCM can provide reliable information on disinfectant efficacy. These findings are consistent with previous studies reporting the suitability of FCM for rapid microbial viability assessment in diverse applications, including water safety and clinical microbiology (13, 30).

A key outcome of this study is the quantitative evaluation of FCM as a predictive tool for bactericidal efficacy. The binary logistic-regression model revealed a strong and statistically significant association between the proportion of PI^+^ cells and bactericidal classification based on the culture-based method (P-value < 0.0001). The model exhibited good discriminatory performance (AUC = 0.787) and enabled the identification of a data-driven threshold of approximately 80% PI^+^ cells for classifying disinfectants as bactericidal. This quantitative criterion represents a practical advantage over the semi-quantitative LogR ≥ 5 reduction endpoint used in standard culture-based assays and supports more standardised and reproducible efficacy assessment (5, 23).

From an applied perspective, the identified PI^+^ threshold provides an actionable benchmark for interpreting FCM results. Rapid, objective assessment of disinfectant performance could facilitate timely adjustments in disinfectant concentration, application frequency or product selection, which is particularly relevant in farm settings where delayed feedback may compromise disease control and worsen economic losses associated with lameness. The high overall agreement between FCM and the culture-based method (92.3%) observed in this study is comparable to that reported in other comparative investigations (15, 23). Importantly, the limited number of discordant results should not be interpreted as methodological shortcomings, but rather as reflections of fundamental differences between the two analytical principles. While dilution–neutralisation assays assess reproductive viability, FCM combined with PI staining evaluates membrane integrity (7). Accordingly, PI uptake should primarily be interpreted as evidence of compromised membrane integrity rather than as definitive proof of cell death. Conversely, the absence of PI staining does not necessarily confirm bacterial viability or culturability, since cells with apparently intact membranes may still be non-culturable or affected by other lethal mechanisms. This distinction is particularly relevant for VBNC cells, a sequela of disinfectant-induced stress (22, 29). Such cells may retain intact membranes and therefore be classified as viable by FCM, despite their inability to form colonies. This retention would explain instances where FCM indicated higher survival than suggested by LogR values. Conversely, extensive PI uptake may indicate severe membrane damage without necessarily corresponding exactly to the loss of culturability measured by EN 1656. These biological differences between membrane integrity and culturability may explain the observed discrepancies and limit the direct use of PI-based FCM as a surrogate for standard bactericidal assays. Therefore, FCM should be regarded as a rapid complementary approach to EN 1656 rather than as a complete replacement.

With respect to disinfectant composition, the efficacy patterns observed in this study are largely consistent with the published literature. Preparations containing iodine, synergistic blends of organic acids or multi-component formulations incorporating glutaraldehyde and/or quaternary ammonium compounds (QACs) demonstrated the highest and most consistent bactericidal activity. The broad-spectrum antimicrobial properties of iodine, glutaraldehyde and QACs are well documented (9, 14), as is the effectiveness of organic acids such as lactic and citric (20, 25). The strong performance of the hydrogen peroxide-based formulation likely reflects synergistic interactions with accompanying components, which may mitigate resistance mechanisms associated with catalase or peroxidase activity (12).

In contrast, disinfectants based on metal salts and formaldehyde exhibited inconsistent or limited efficacy. Zinc sulphate was ineffective against all tested strains, while copper sulphate showed reduced activity against Gram-positive bacteria, potentially because of known resistance mechanisms such as the *cop* operon in staphylococci and the *tcr* gene in enterococci (24). In addition, the relatively short contact time specified by EN 1656 may be insufficient to fully elicit the oligodynamic effects of heavy metals, which often require prolonged exposure (28). The limited efficacy of formaldehyde, particularly against Gram-positive bacteria, is consistent with current understanding of its antimicrobial mode of action (16).

Bacterial susceptibility to disinfectants was clearly species dependent. In general, the Gram-negative bacteria *Pseudomonas aeruginosa* and *Proteus hauseri* were more susceptible than Gram-positive cocci, a finding that both aligns with and challenges traditional assumptions regarding intrinsic resistance associated with the Gram-negative outer membrane (4). The pronounced resistance of *Enterococcus hirae* supports its suitability as a robust reference organism for disinfectant efficacy testing. Separately, the consistent susceptibility of *P. aeruginosa* is particularly relevant to farm environments, given its genetic similarities to *Dichelobacter nodosus*, a key pathogen involved in infectious hoof diseases (11).

Several limitations of this study should be acknowledged. All experiments were conducted under controlled laboratory conditions without organic load, which may lead to an overestimation of disinfectant efficacy compared with field conditions (19). In addition, the use of reference bacterial strains may not fully reflect the diversity and resistance profiles of field isolates. Although the predictive model demonstrated good performance using a limited dataset and a single predictor, future studies incorporating larger datasets, additional predictors and dual-staining approaches could further refine FCM-based efficacy assessment. Nevertheless, the present findings provide a robust foundation for the application of flow cytometry as a quantitative screening tool for disinfectant evaluation in veterinary practice. The rapidity of the method’s results recommend it as a complement to periodic laboratory testing, and offers the potential for more dynamic adjustment of disinfection protocols based on near real-time disinfectant performance data.

## Conclusion

This study demonstrates that flow cytometry is a rapid, complementary and quantitative laboratory-based screening method for assessing disinfectant efficacy in cattle footbath formulations, using membrane damage as an alternative endpoint to the post-neutralisation viability in the standardised EN 1656 culture-based approach. The developed binary logistic regression model showed good predictive performance and enabled the identification of a data-driven threshold of approximately 80% PI^+^ cells for classifying bactericidal activity, providing a practical quantitative benchmark for laboratory evaluation of disinfectant efficacy. However, because PI uptake primarily reflects loss of membrane integrity rather than culturability, this threshold should be interpreted as a supportive screening criterion rather than as a direct replacement for the EN 1656 endpoint.

The results revealed substantial variability in the performance of commercially available footbath disinfectants, underscoring the importance of evidence-based product selection. While multi-component formulations containing iodine, glutaraldehyde, quaternary ammonium compounds or organic acids demonstrated consistently high efficacy, disinfectants based on metal salts exhibited limited or insufficient bactericidal activity under the test conditions.

By substantially reducing the time required for efficacy assessment compared with culture-based methods, FCM offers a promising complementary tool for improving laboratory-based evaluation of disinfectants used in veterinary practice. The adoption of this approach has the potential to support more informed decision-making in footbath management and contribute to improved biosecurity strategies aimed at controlling infectious hoof diseases. Future research should focus on validating this screening approach under simulated and field-relevant ‘dirty’ conditions, and on incorporating a broader range of bacterial isolates to further assess its applicability as a complementary method to standardised culture-based efficacy testing.

## References

[1] Ariza J.M., Relun A., Bareille N., Oberle K., Guatteo R. (2017). Effectiveness of collective treatments in the prevention and treatment of bovine digital dermatitis lesions: A systematic review. J Dairy Sci.

[2] Berney M., Hammes F., Bosshard F., Weilenmann H.U., Egli T. (2007). Assessment and interpretation of bacterial viability by using the LIVE/DEAD BacLight Kit in combination with flow cytometry. Appl Environ Microbiol.

[3] Boulos L., Prévost M., Barbeau B., Coallier J., Desjardins R. (1999). LIVE/DEAD BacLight: application of a new rapid staining method for direct enumeration of viable and total bacteria in drinking water. J Microbiol Methods.

[4] Brown M.R., Gilbert P. (1993). Sensitivity of biofilms to antimicrobial agents. J Appl Bacteriol.

[5] Claveau L., Hudson N., Jarvis P., Jeffrey P., Hassard F. (2024). Microbial water quality investigation through flow cytometry fingerprinting: from source to tap. Sustain Microbiol.

[6] Cook N.B., Rieman J., Gomez A., Burgi K. (2012). Observations on the design and use of footbaths for the control of infectious hoof disease in dairy cattle. Vet J.

[7] Ersoy Z.G., Dinc O., Cinar B., Gedik S.T., Dimoglo A. (2019). Comparative evaluation of disinfection mechanism of sodium hypochlorite, chlorine dioxide and electroactivated water on *Enterococcus faecalis*. LWT.

[8] European Committee for Standardization (CEN) (2020). European Standard EN 1656:2020 Chemical disinfectants and antiseptics–Quantitative suspension test for the evaluation of bactericidal activity of chemical disinfectants and antiseptics used in the veterinary area–Test methods and requirements (phase 2, step 1).

[9] Gerba C.P. (2015). Quaternary ammonium biocides: efficacy in application. Appl Environ Microbiol.

[10] Grootaert C., Boon N., Zeka F., Vanhoecke B., Bracke M., Verstraete W., Van de Wiele T. (2011). Adherence and viability of intestinal bacteria to differentiated Caco-2 cells quantified by flow cytometry. J Microbiol Methods.

[11] Johnston J.L., Billington S.J., Haring V., Rood J.I. (1998). Complementation analysis of the *Dichelobacter nodosus*
*fimN, fimO*, and *fimP* genes in *Pseudomonas aeruginosa* and transcriptional analysis of the *fimNOP* gene region. Infect Immun.

[12] Lambert P.A. (2002). Cellular impermeability and uptake of biocides and antibiotics in gram-positive bacteria and mycobacteria. J Appl Microbiol.

[13] Lindivat M., Bratbak G., Larsen A., Hess-Erga O.K., Hoell I.A. (2021). Flow cytometric analysis of bacterial protein synthesis: monitoring vitality after water treatment. Front Microbiol.

[14] Maillard J.Y., Pascoe M. (2024). Disinfectants and antiseptics: mechanisms of action and resistance. Nat Rev Microbiol.

[15] Massicotte R., Mafu A.A., Ahmad D., Deshaies F., Pichette G., Belhumeur P. (2017). Comparison between flow cytometry and traditional culture methods for efficacy assessment of six disinfectant agents against nosocomial bacterial species. Front Microbiol.

[16] McDonnell G.E. (2020). Antisepsis, disinfection, and sterilization: Types, action, and resistance.

[17] McFeters G.A., Egli T., Wilberg E., Alder A., Schneider R., Suozzi M., Giger W. (1990). Activity and adaptation of nitrilotriacetate (NTA)-degrading bacteria: field and laboratory studies. Water Res.

[18] O'Hara C.M., Brenner F.W., Steigerwalt A.G., Hill B.C., Holmes B., Grimont P.A.D., Hawkey P.M., Penner J.L., Miller J.M., Brenner D.J. (2000). Classification of *Proteus vulgaris* biogroup 3 with recognition of *Proteus hauseri* sp. nov., nom. rev. and unnamed *Proteus* genomospecies 4, 5 and 6. Int J Syst Evol Microbiol.

[19] Ölmez H., Kretzschmar U. (2009). Potential alternative disinfection methods for organic fresh-cut industry for minimising water consumption and environmental impact. LWT.

[20] Park S.H., Choi M.R., Park J.W., Park K.H., Chung M.S., Ryu S., Kang D.H. (2011). Use of organic acids to inactivate *Escherichia coli* O157:H7, *Salmonella* Typhimurium, and *Listeria monocytogenes* on organic fresh apples and lettuce. J Food Sci.

[21] Park S.Y., Kim C.G. (2018). A comparative study of three different viability tests for chemically or thermally inactivated *Escherichia coli*. Environ Eng Res.

[22] Pazos-Rojas L.A., Cuellar-Sánchez A., Romero-Cerón A.L., Rivera-Urbalejo A., Van Dillewijn P., Luna-Vital D.A., Muñoz-Rojas J., Morales-García Y.E. (2023). The viable but non-culturable (VBNC) state, a poorly explored aspect of beneficial bacteria. Microorganisms.

[23] Pîndaru A.M., Măruţescu L., Popa M., Chifiriuc M.C. (2024). A label-free optical flow cytometry based-method for rapid assay of disinfectants’ bactericidal activity. Int J Mol Sci.

[24] Rosario-Cruz Z., Eletsky A., Daigham N.S., Al-Tameemi H., Swapna G.V.T., Kahn P.C., Szyperski T., Montelione G.T., Boyd J.M. (2019). The *copBL* operon protects *Staphylococcus aureus* from copper toxicity: CopL is an extracellular membrane-associated copper-binding protein. J Biol Chem.

[25] Seo Y.S., Lee G., Song S., Kim K., Cho M. (2021). Combinatorial treatment using citric acid, malic acid, and phytic acid for synergistic inactivation of foodborne pathogenic bacteria. Korean J Chem Eng.

[26] Solano L., Barkema H.W., Pickel C., Orsel K. (2017). Effectiveness of a standardised footbath protocol for prevention of digital dermatitis. J Dairy Sci.

[27] Wang Y., Hammes F., De Roy K., Verstraete W., Boon N. (2010). Past, present and future applications of flow cytometry in aquatic microbiology. Trends Biotechnol.

[28] Wilks S.A., Michels H., Keevil C.W. (2005). The survival of *Escherichia coli* O157 on a range of metal surfaces. Int J Food Microbiol.

[29] Ye C., Chen C., Feng M., Ou R., Yu X. (2024). Emerging contaminants in the water environment: Disinfection-induced viable but non-culturable waterborne pathogens. J Hazard Mater.

[30] Zdeb M., Zamorska J., Domon A. (2018). Disinfection of rainwater as a way to their microbiological stability and safe use. E3S Web Conf.

